# *In vivo* immune signatures of healthy human pregnancy: Inherently inflammatory or anti-inflammatory?

**DOI:** 10.1371/journal.pone.0177813

**Published:** 2017-06-21

**Authors:** Caroline Graham, Rishma Chooniedass, William P. Stefura, Allan B. Becker, Malcolm R. Sears, Stuart E. Turvey, Piush J. Mandhane, Padmaja Subbarao, CHILD Study Investigators, Kent T. HayGlass

**Affiliations:** 1 Department of Immunology, University of Manitoba, Winnipeg, Manitoba, Canada; 2 Department of Pediatrics and Child Health, University of Manitoba, Winnipeg, Manitoba, Canada; 3 Children’s Hospital Research Institute of Manitoba, Winnipeg, Manitoba, Canada; 4 Department of Medicine, de Groote School of Medicine, McMaster University, Hamilton, Ontario, Canada; 5 Department of Pediatrics, Child & Family Research Institute and BC Children’s Hospital, University of British Columbia, Vancouver, British Columbia, Canada; 6 Department of Pediatrics, University of Alberta, Edmonton, Alberta, Canada; 7 Department of Pediatrics, Hospital for Sick Children, University of Toronto, Toronto, Ontario, Canada; 8 CHILD (Canadian Healthy Infant Longitudinal Development Study) Investigators, McMaster University, Hamilton, Canada; Indiana University, UNITED STATES

## Abstract

Changes in maternal innate immunity during healthy human pregnancy are not well understood. Whether basal immune status *in vivo* is largely unaffected by pregnancy, is constitutively biased towards an inflammatory phenotype (transiently enhancing host defense) or exhibits anti-inflammatory bias (reducing potential responsiveness to the fetus) is unclear. Here, in a longitudinal study of healthy women who gave birth to healthy infants following uncomplicated pregnancies within the Canadian Healthy Infant Longitudinal Development (CHILD) cohort, we test the hypothesis that a progressively altered bias in resting innate immune status develops. Women were examined during pregnancy and again, one and/or three years postpartum. Most pro-inflammatory cytokine expression, including CCL2, CXCL10, IL-18 and TNFα, was reduced *in vivo* during pregnancy (20–57%, p<0.0001). Anti-inflammatory biomarkers (sTNF-RI, sTNF-RII, and IL-1Ra) were elevated by ~50–100% (p<0.0001). Systemic IL-10 levels were unaltered during vs. post-pregnancy. Kinetic studies demonstrate that while decreased pro-inflammatory biomarker expression (CCL2, CXCL10, IL-18, and TNFα) was constant, anti-inflammatory expression increased progressively with increasing gestational age (p<0.0001). We conclude that healthy resting maternal immune status is characterized by an increasingly pronounced bias towards a systemic anti-inflammatory innate phenotype during the last two trimesters of pregnancy. This is resolved by one year postpartum in the absence of repeat pregnancy. The findings provide enhanced understanding of immunological changes that occur *in vivo* during healthy human pregnancy.

## Introduction

For healthy pregnancy to proceed to term, changes need to occur to prevent immune mediated rejection of the semi-allogenic fetus. At the same time, the immune system must maintain, or enhance, protection of mother and fetus from external pathogens. There is extensive literature concerning immunity at the maternal-fetal interface and its role in progression of fetal development [1–5]. Similarly, many studies have examined pathologic conditions that can arise during pregnancy (i.e. preeclampsia, infection, hypoxia), often with small, cross-sectional, healthy control groups for comparison.

Surprisingly, healthy pregnancy that leads to healthy infants has not been a major research focus and is not well understood. Publications [6] and recent NIH workshops [7, 8] identify the need for better insight into the biology of normal pregnancy. Attention needs to be given to (i) understanding maternal adaptations and (ii) creating a biological definition of an optimal pregnancy phenotype from fetal, maternal and paternal standpoints. Understanding putative changes in women’s *in vivo* innate immune status during normal pregnancy–the healthy phenotype–will strengthen efforts to understand linkages between *in vivo* maternal status and the subsequent development of healthy vs. chronic inflammatory phenotypes such as asthma or autoimmunity in children, or their mothers, later in life [9–13].

The changes, if any, that occur in innate immune status during a healthy pregnancy are controversial. Existing evidence supports several mutually exclusive conclusions. Some data are consistent with the concept that basal maternal systemic immunity exhibits a mild bias towards inflammatory phenotypes (hence, transiently enhancing host defense). Others support the notion that immunosuppressed phenotypes (reducing potential responses to the fetus) are normally dominant during pregnancy [14]. A third school of thought argues that innate immune function is largely unchanged in pregnant and non-pregnant women [3, 15].

Interest in healthy pregnancy is driven by at least three other rationales. Exclusion of pregnant women from clinical research, while well intentioned, can be counterproductive [7]. Their inclusion requires better understanding of maternal health norms during healthy pregnancy. Secondly, identifying and understanding differences in basal innate immune status *in vivo* during healthy pregnancy will provide better understanding of mechanisms that underlie difficult pregnancies [15]. Finally, with extensive efforts to link systemic innate immunity *in vivo* and clinical outcomes later in life for both mother and fetus [16, 17], we need to better define immunity in healthy human pregnancy as the entry point to childhood.

The Canadian Healthy Infant Longitudinal Development (CHILD) birth cohort was initiated to study the development of allergy and asthma, with a strong focus on clinical, immunologic and environmental assessments of infants and parents. The 3,624 participating infants and families are predominantly from urban centres; over 80% of the Canadian population is urban. The cohort is multicultural and ethnically varied. Its value is enhanced by extensive phenotyping of both children and parents, characterization of their environments and an extensive repository of biological samples [18].

Here we test the hypothesis that resting systemic pro-/anti-inflammatory bias *in vivo* is transiently shifted during pregnancy. In this longitudinal study of 251 randomly selected healthy women who gave birth to healthy infants, pairwise comparisons were used to assess innate immune biomarker levels *in vivo* during the second/third trimester then again at one and three years postpartum. An extensive panel of pro-inflammatory cytokines that are constitutively present in most healthy individuals (CCL2, CXCL10, CXCL8, IL-18, IL-6, and TNFα) was examined. While studies of inflammatory processes often include few or no anti-inflammatory regulators, endogenous levels of a broad panel of anti-inflammatory cytokines (IL-10, IL-1Ra, sTNF-RI, and sTNF-RII) were incorporated to provide a better immune signature of women’s health during successful pregnancy. The data reveal that extensive changes occur *in vivo*. Healthy pregnancy is linked to development of a constitutive systemic anti-inflammatory maternal bias that becomes increasingly intense with increasing gestational age, then self-resolves by one year postpartum.

## Materials and methods

### Participants

The Canadian Healthy Infant Longitudinal Development (CHILD) Study is a prospective national population-based longitudinal birth cohort of 3,624 neonates (http://www.canadianchildstudy.ca/knowledge.html) [18]. Following written, informed consent, non-fasting venous blood was obtained at the University of Manitoba recruitment site to yield plasma samples from 499 women. Median age at recruitment was 31.1 years. Women were recruited between conception and delivery, as possible, with most recruitment taking place during the second and third trimester. Inclusion criteria included uneventful pregnancy without documented concerns about hypertension, proteinuria or gestational diabetes that resulted in a healthy singleton baby. Exclusion criteria included in vitro fertilization (IVF), twins, miscarriage, intrauterine growth restriction (IUGR), clinically discernible upper respiratory tract (URT) or gastrointestinal (GI) infection within a week of study visit or repeat pregnancy evident at or within two months following one year and three year postpartum visits. This study was approved by the University of Manitoba Human Research Ethics Board.

To increase power, rather than incorporating unrelated non-pregnant female controls, a longitudinal study design was used where a woman’s status during pregnancy was compared with her own at subsequent times. Samples were obtained once at initial recruitment (a visit between gestational weeks 10–38) and again one year postpartum. Major clinical characteristics of the women studied are provided in Table 1.

**Table 1:** Demographic and clinical characteristics of study population.

No. of Women in Study	251
Maternal Age at Delivery (years)	31.1 (18.4–43.2)
Gestational Age at PN Visit (weeks)	27.0 (9.9–38.4)
Gestational Age at Delivery (weeks)	38.0 (35.0–42.1)
Maternal Tobacco Use	5%
Preeclampsia	0% (excluded)
Gestational Diabetes Mellitus (GDM)	0% (excluded)

Values are presented as median (range) or as a percentage of the total population.

251 of the mothers for whom paired plasma samples were available from prenatal (PN) and one year post-partum (1YPP) time points (n = 331) were randomly selected for analysis of *in vivo* pro- and anti-inflammatory cytokine expression (CCL2 and sTNF-RI). These biomarkers were chosen because published, and our own preliminary data, demonstrated that readily quantified levels are evident in >95% of healthy individuals (cf. IL-6 or IL-10 where a substantial proportion of healthy individuals exhibit sub pg/ml plasma levels). For more extensive analyses, 8 additional *in vivo* biomarkers were determined using approximately every second individual (n = 120) from the original study group. Investigators were blind to any immunological data at the time of randomization and sample selection.

Among women who provided plasma samples at both one and three years post-partum, (and who did not experience pregnancy in the interim) 32 were randomly selected to further compare potential changes in selected plasma biomarker levels post-pregnancy (see Results).

### Immunological assays

Samples were processed as described [19]. Briefly, whole blood samples were kept at room temperature during transport and prior to processing later that day. Whole blood plasma was collected from 10 mL sodium heparin Vacutainer tubes (BD, Mississauga, Canada) by centrifugation at 500g for 10 minutes, aliquoted and stored at -20°C. Immediately prior to use in immunological assays, thoroughly mixed plasma was subjected to a quick spin (500g, 1 minute) to remove protein/lipid precipitate. Samples for pregnant and one year postpartum, or one and three years postpartum, were analyzed as pairs on the same assay plates.

Meso Scale Discovery (MSD, Rockville, Maryland) singleplex assays were used to quantify analyte concentrations following the manufacturer`s instructions. Catalog numbers for the reagent kits used are provided at Table 2.

**Table 2:** Catalog numbers for Meso Scale Discovery kits used in this publication.

Cytokine	MSD Catalog Number
CCL2	K151AYB-2
CXCL8	K151ANB-2
CXCL10	K151NVD-2
IL-6	K151AKB-2
IL-10	K151AOB-2
IL-18	K151MCD-2
IL-1Ra	N45ZA-1
sTNF-RI	K151BIC-2
sTNF-RII	K151BJB-2
TNFα	K151BHB-2

For all assays, concentrations were determined based on standard curves created using serial dilutions of fresh aliquots of constant recombinant lab standards prepared in culture medium and stored at -80°C in individual 400 uL aliquots (Cedarlane, Burlington, Canada; PeproTech, Quebec, Canada; R&D Systems, Minneapolis, Minnesota). Longitudinal sample sets from each individual were always paired on the same assay plates. Median intra-assay variation was typically below 5%.

### Statistics

Group results are presented as medians, with each point representing the average (mean) value obtained from duplicate or triplicate analyses of an individual woman’s plasma at that timepoint. Supporting information S1 File contains the raw duplicates or triplicates used to calculate the mean values for each cytokine or chemokine, for each woman, at each visit, under each condition examined, that were then used to create the figures presented. Data for each population were analyzed using GraphPad Prism (La Jolla, California). Pairwise comparisons (Wilcoxon Matched Pairs/Signed Rank tests) were used for most data sets. The Spearman rank order correlation coefficient (non-parametric) was used to assess potential associations between plasma biomarker levels and increasing gestational age. Differences were considered significant at the 95% confidence level (two-tailed p<0.05).

## Results

### Alterations in basal innate immune balance are characteristic of healthy human pregnancy

**Fig 1:**
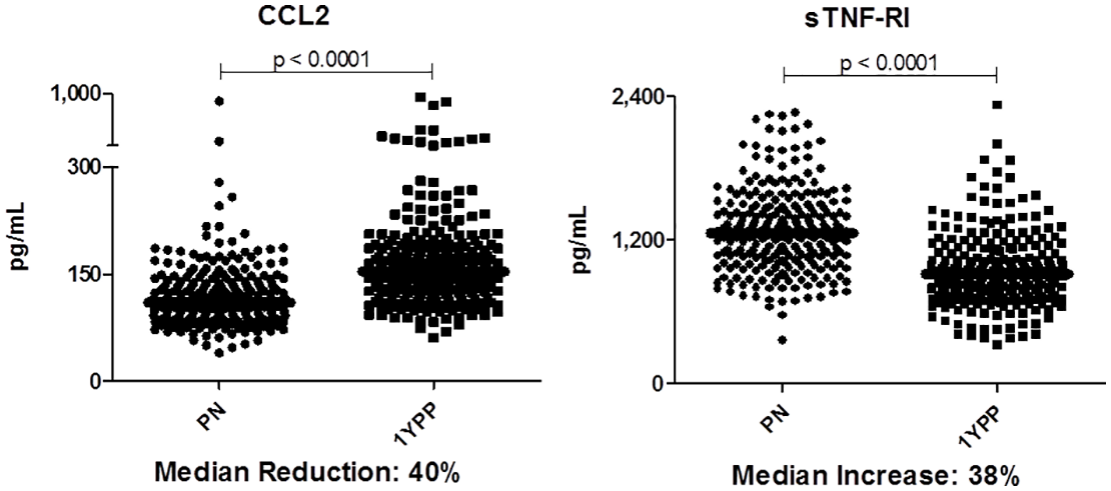
Among healthy women, a systemic bias towards a resting anti-inflammatory phenotype is evident *in vivo* during pregnancy. Paired plasma samples from 251 women are assessed during and one year following healthy pregnancy. Data are presented as pg/mL. Bars represent median values of each data set. Wilcoxon paired analyses are shown.

This longitudinal study aimed to determine whether the constitutive immune status of healthy pregnant women exhibits a pro- or anti-inflammatory bias and whether it changes during pregnancy. As described in detail at Material and Methods, plasma samples were obtained at a single time point during pregnancy, again one year postpartum, and for a randomly obtained subset of individuals who did not experience further pregnancies in the interim, at three years postpartum. Two representative biomarkers of pro- and anti-inflammatory responses were assessed initially (Fig 1). Pro-inflammatory chemokine CCL2 was readily detectible in all individuals. Among the 251 women examined, systemic CCL2 was found to be reduced by 40% during pregnancy compared to levels one year postpartum (p<0.0001, Wilcoxon matched pairs, medians 110 vs. 154 pg/mL). Conversely, sTNF-RI was 38% elevated during pregnancy (medians 1,266 vs. 913, p<0.0001). Thus, the median ratio of these anti-inflammatory:pro-inflammatory innate biomarkers (sTNF-RI:CCL2) changed from 11.4 during pregnancy to 5.9 a year following delivery. This suggests that constitutive maternal status may incline towards an anti-inflammatory balance over the course of pregnancy.

Given the large number of pro- and anti-inflammatory cytokines and inhibitors involved in human immune regulation, this analysis was extended in approximately every second woman (n = 120) to a panel of five additional pro-inflammatory biomarkers (CXCL10, TNFα, IL-18, IL-6 and CXCL8) as well as three more anti-inflammatory biomarkers (IL-10, sTNF-RII and IL-1Ra). The cytokines selected were chosen on the basis of being (i) routinely present in the plasma of healthy humans and (ii) having been extensively implicated in immune regulation of inflammation in murine and human studies. These large panels of biomarkers were used in preference to relying upon a single biomarker (CRP or IL-6 or IL-10, as is commonly done) in order to strengthen the capacity to draw conclusions about pro- vs. anti- inflammatory bias *in vivo* during pregnancy. A brief overview of the major cellular sources and biological functions of the cytokines, chemokines and receptors studied is provided in Table 3.

**Table 3:** Cytokine, chemokine and soluble receptor biomarkers examined in this publication.

Cytokine	Major Sources	Major Functions	Major role as a Pro- or Anti-Inflammatory immune response modifier
CCL2 (MCP-1)	Macrophages, monocytes, endothelial cells, fibroblasts, epithelial cells, smooth muscle cells, mesangial cells, astrocytic cells, monocytic and microglial cells [20].	CCL2 recruits monocytes, memory T cells, and dendritic cells to sites of inflammation [21–23]. It has been Implicated in pathogenesis or exacerbation of many inflammatory diseases including asthma, atherosclerosis, gestational diabetes mellitus, hypertension and rheumatoid arthritis [24, 25].	Pro-Inflammatory
CXCL8 (IL-8)	Macrophages, monocytes, epithelial cells, airway smooth muscle cells and endothelial cells [26, 27].	CXCL8 is an inflammatory chemokine involved in neutrophil (and other granulocyte) recruitment/chemotaxis towards sites of infection and subsequent phagocytosis/ degranulation [27]. It is also a potent promoter of angiogenesis [28–30].	Pro-Inflammatory
CXCL10 (IP-10)	Macrophages, monocytes, endothelial cells and fibroblasts [31].	CXCL10 is involved in chemoattraction for monocytes/ macrophages, T cells, NK cells, and dendritic cells [32, 33]. It is also involved in the promotion of T cell adhesion to endothelial cells, enhances Th1 activity and has antitumor activity [32–35].	Pro-Inflammatory
IL-6	Macrophages, monocytes, T cells, endothelial cells, placental cells and adipocytes [20, 35, 36].	In response to infection (e.g. viruses, bacteria) or trauma (e.g. surgery, burns, tissue damage), IL-6 is secreted and stimulates inflammation [37]. It is an important mediator of fever, the acute phase response, neutrophil production in bone marrow, supports B cell growth and is antagonistic to regulatory T cells [35]. Clinically, it stimulates inflammatory processes in host defense and many diseases including diabetes [38], atherosclerosis [39], depression [40], Alzheimer’s [41], systemic lupus erythematosus [42], multiple myeloma [43], prostate cancer [44], and rheumatoid arthritis [45].	Pro-Inflammatory
IL-10	Macrophages, monocytes, T cells, B cells, NK cells, mastocytes and adipocytes [20, 46, 47].	IL-10 inhibits T cell derived cytokine production, MHC class II and co-stimulatory molecule expression, and enhances B cell survival, proliferation, and antibody production [48–52]. It inhibits TLR mediated induction of multiple pro-inflammatory cytokines including TNFα, IL-1β, IL-12, and IFNγ [46, 47].	Anti-Inflammatory
IL-18	Macrophages, monocytes, dendritic cells, endothelial cells, keratinocytes and intestinal epithelial cells [53, 54].	IL-18 induces cell-mediated immunity following infection with microbial products (e.g. LPS) [53]. IL-18 stimulation of NK and T cells stimulates IFNγ release, activating macrophages and other cells [55–57].	Pro-Inflammatory
IL-1Ra	Macrophages, monocytes, neutrophils, mast cells, epithelial cells and adipocytes [46, 54, 58–60].	IL-1Ra inhibits the activities of pro-inflammatory cytokines IL-1α and IL-1β, modulating IL-1 related immune and inflammatory responses [58, 61–63].	Anti-Inflammatory
sTNF-RI	Macrophages, monocytes, T cells, B cells, NK cells, dendritic cells, endothelial cells and epithelial cells [46, 54, 64].	sTNF-RI is generated by the shedding of its membrane-expressed counterpart (TNF-RI) [65]. Thus, sTNF-RI has inherent anti-inflammatory activity, as it competes with membrane-associated receptors for the binding of free cytokines, confining the pro-inflammatory cytokine activity to the local site of inflammation [65, 66].	Anti-Inflammatory
sTNF-RII	Macrophages, monocytes, T cells, B cells, NK cells, dendritic cells, endothelial cells and epithelial cells [46, 54, 64].	sTNF-RII is generated via shedding of a membrane-expressed counterpart (TNF-RII) [65]. As for sTNF-RI, it competes with membrane-associated signaling receptors for the TNF binding, confining the pro-inflammatory cytokine activity to the local site of inflammation [65, 66], it has inherent anti-inflammatory activity.	Anti-Inflammatory
TNFα	Macrophages, monocytes, T cells, NK cells, neutrophils, mast cells, eosinophils and neurons [20, 67].	TNFα is a major player in chronic and acute inflammation [67, 68]. It also inhibits tumorigenesis and viral replication [67]. TNFα dysregulation has been implicated in a broad variety of human inflammatory diseases [68, 69].	Pro-Inflammatory

**Fig 2:**
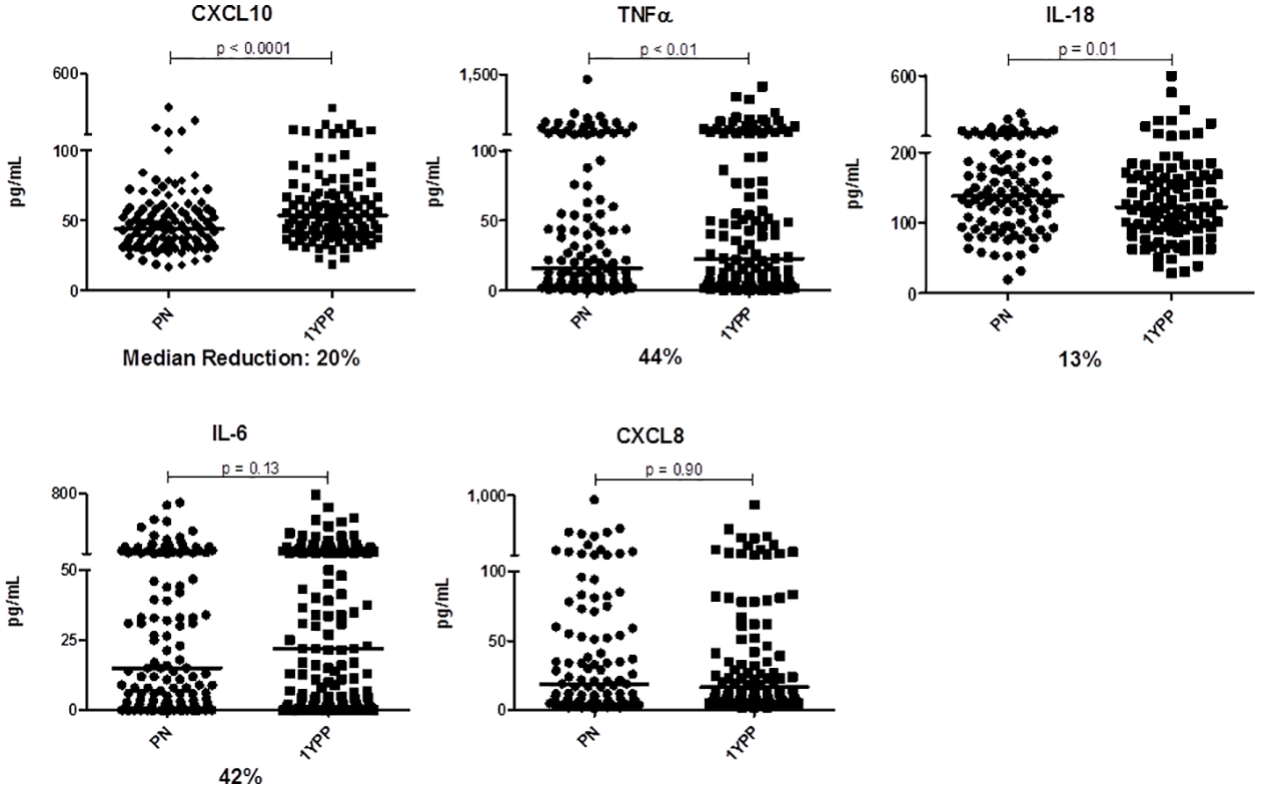
Basal levels of multiple pro-inflammatory innate biomarkers are reduced *in vivo* during healthy pregnancy. n = 120 longitudinal sample pairs. Bars represent median values of each data set. Wilcoxon paired analyses are shown.

Fig 2 demonstrates that during healthy pregnancy, most pro-inflammatory cytokines were indeed reduced relative to levels in the same women one year postpartum. Thus, constitutive CXCL10, TNFα and IL-18 were reduced during pregnancy. Median IL-6 trended lower by 42%, but the difference did not reach statistical significance. There was no evidence of changes in constitutive CXCL8 levels during healthy pregnancy. Note that due to the inherent immunological diversity characteristic of human populations, and to avoid compression of clearly positive but low levels of cytokine expression in some individuals, y-axis breaks are included.

**Fig 3:**
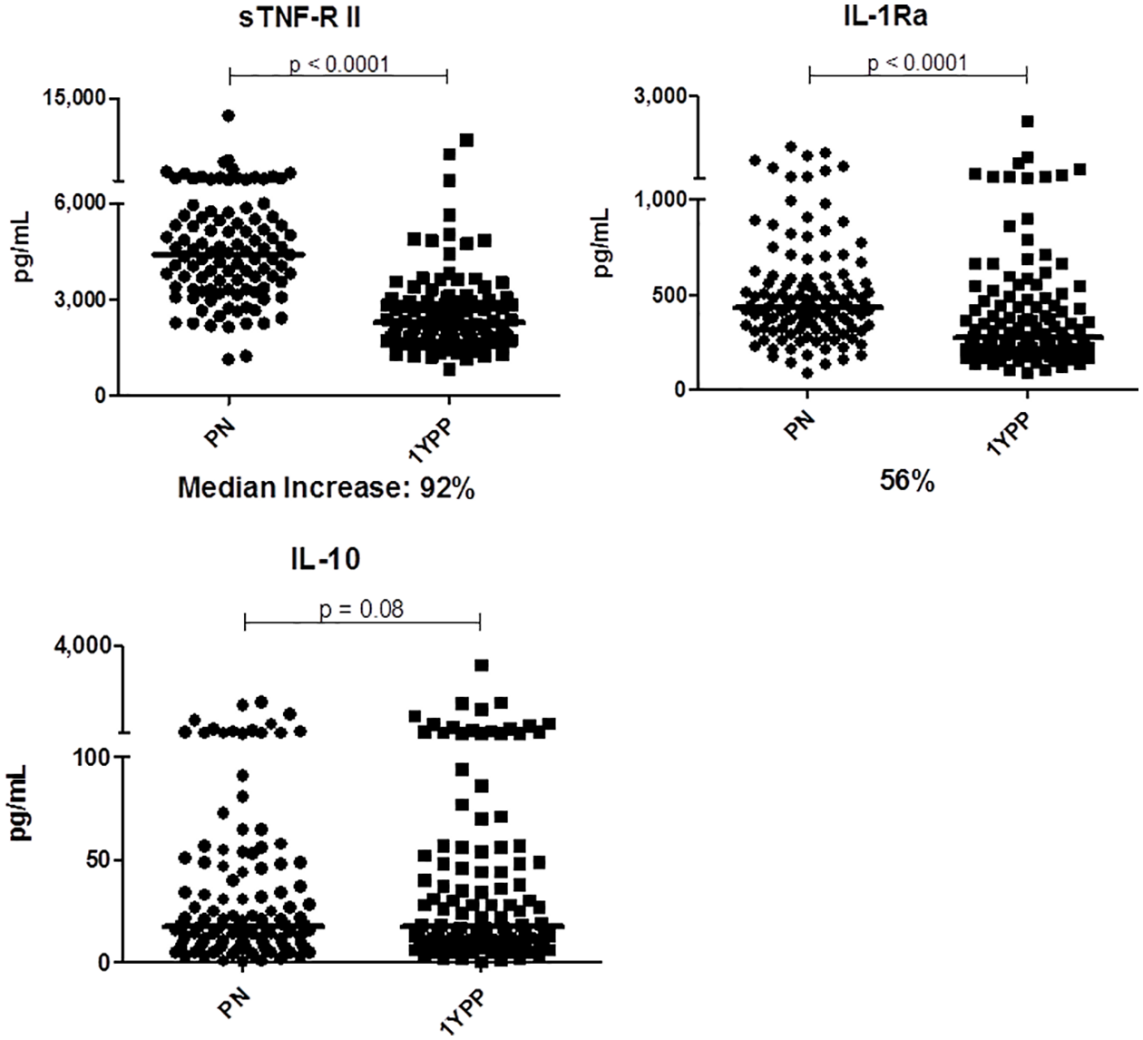
Constitutive expression of multiple anti-inflammatory biomarkers is increased during pregnancy. n = 120 longitudinal sample pairs. Bars represent median values of each data set. Wilcoxon paired analyses are shown.

Anti-inflammatory biomarkers (sTNF-RI, sTNF-RII, and IL-1Ra) demonstrated that unlike the reductions seen in constitutive pro-inflammatory cytokine expression, anti-inflammatory biomarker levels were elevated during pregnancy (Figs 1 and 3, p<0.0001). Interestingly, plasma IL-10, readily quantified in ~85% of the population, did not differ. Taken together, the data suggest that healthy pregnancy is characterized by transiently decreased pro-, and increased anti-inflammatory expression. This self-resolves by one year postpartum.

### Validity of one year postpartum as a surrogate for the non-pregnant state

**Fig 4:**
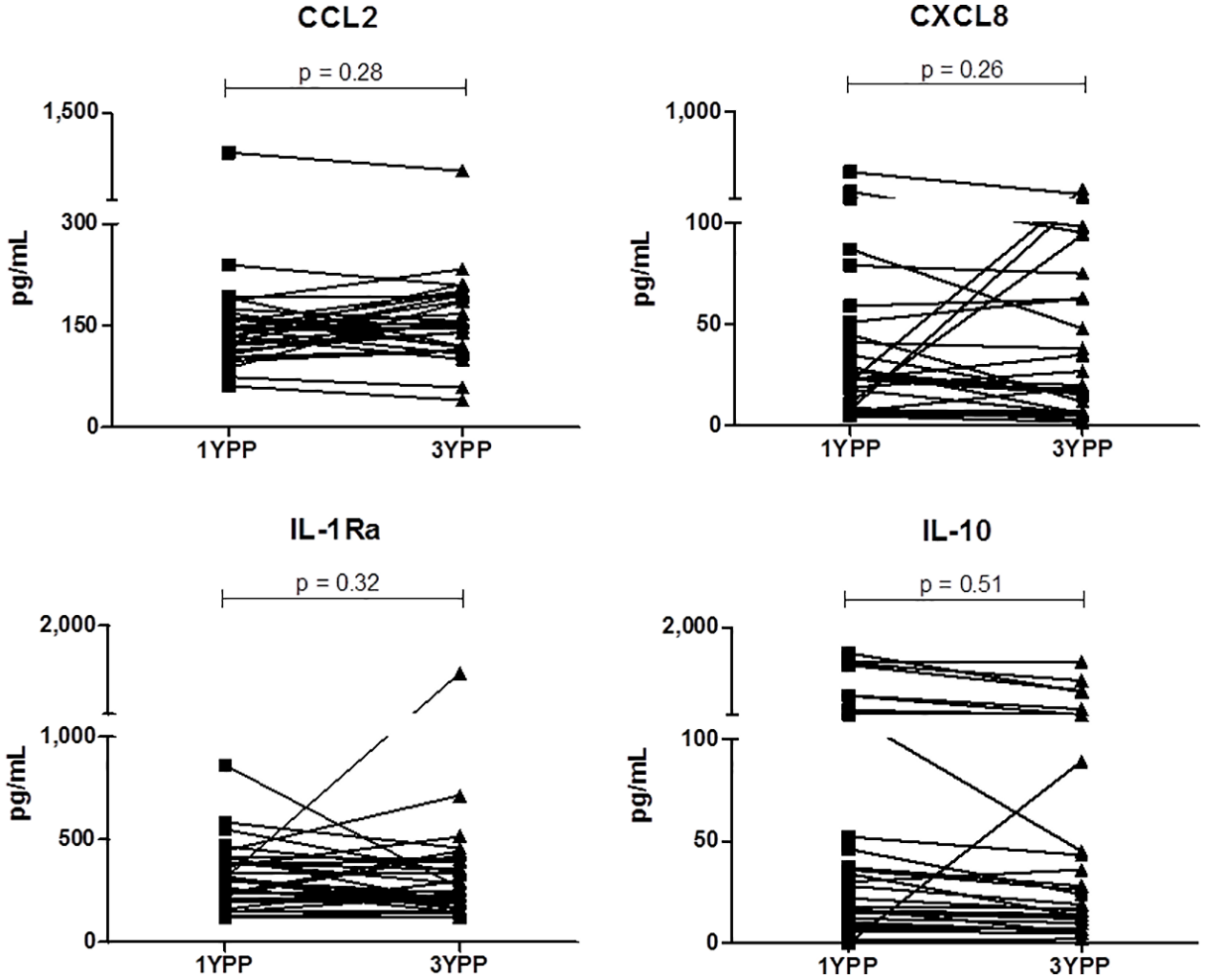
Stability of *in vivo* pro- and anti-inflammatory plasma biomarkers. n = 32 longitudinal sample pairs. Wilcoxon paired analyses are shown.

It is not logistically feasible to recruit women one year prior to an anticipated conception in order to assess their then basal systemic immune status. We therefore addressed the possibility that one year postpartum does not allow sufficient time for the innate immune system to return to the non-pregnant status, hence is not an appropriate comparison group. 32 randomly selected women who remained non-pregnant over the two years following the one year postpartum visit were examined. At this year three visit, plasma samples were obtained and compared (at the same time, in parallel assays) with plasma obtained from the same women at one year postpartum. One pro- and one anti-inflammatory biomarker that had exhibited differential expression during pregnancy (CCL2 and IL-1Ra) and one pro- and one anti-inflammatory biomarker that had been unchanged during pregnancy (CXCL8 and IL-10) were examined. Fig 4 demonstrates that the level of each cytokine was stable within an individual at one and three years post-pregnancy. This supports the concept that one (or three) year postpartum samples offer an appropriately stable comparator for non-pregnant status in this longitudinal study.

### Anti-inflammatory bias is evident at least as early as the second trimester

**Fig 5:**
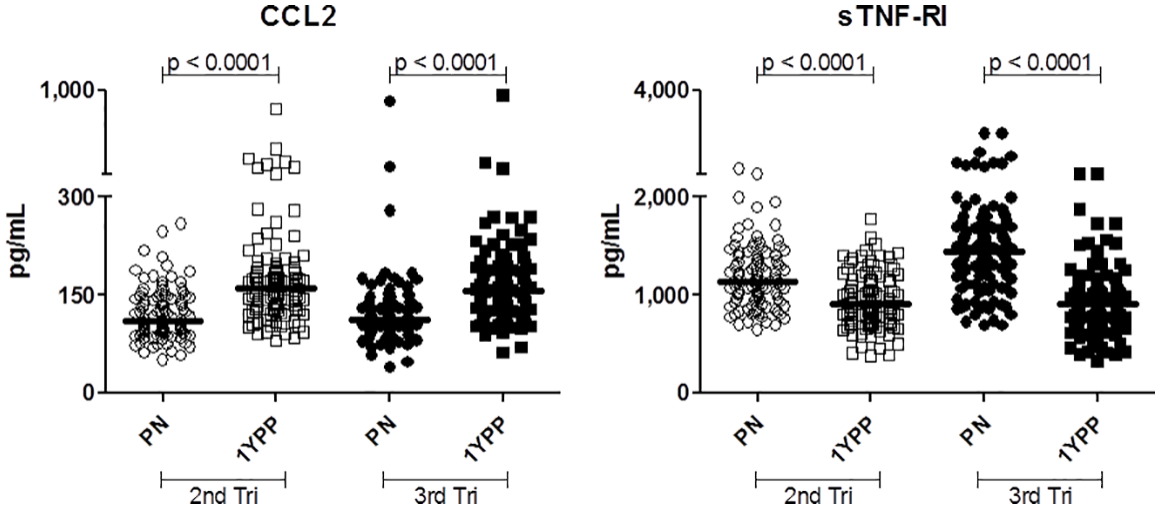
Longitudinal analysis of differences in pro- and anti-inflammatory cytokines in second and third trimesters. Independent panels of volunteers were examined during the second or third trimester and compared with levels one year postpartum. Bars represent median values of each data set. Wilcoxon paired analyses are shown.

The comparisons above test the hypothesis that resting constitutive innate immune status is distinct during healthy pregnancy. To better understand the kinetics of this shift in resting immune status, the population was next stratified by trimester of recruitment. With only a small number of women recruited during the first trimester, analysis centred on the second and third trimesters. Fig 5 demonstrates basal (i.e. healthy, resting) innate immune status is skewed towards an anti-inflammatory phenotype at least as early as the second trimester.

### The intensity of anti-inflammatory bias increases with increasing gestational age

**Fig 6:**
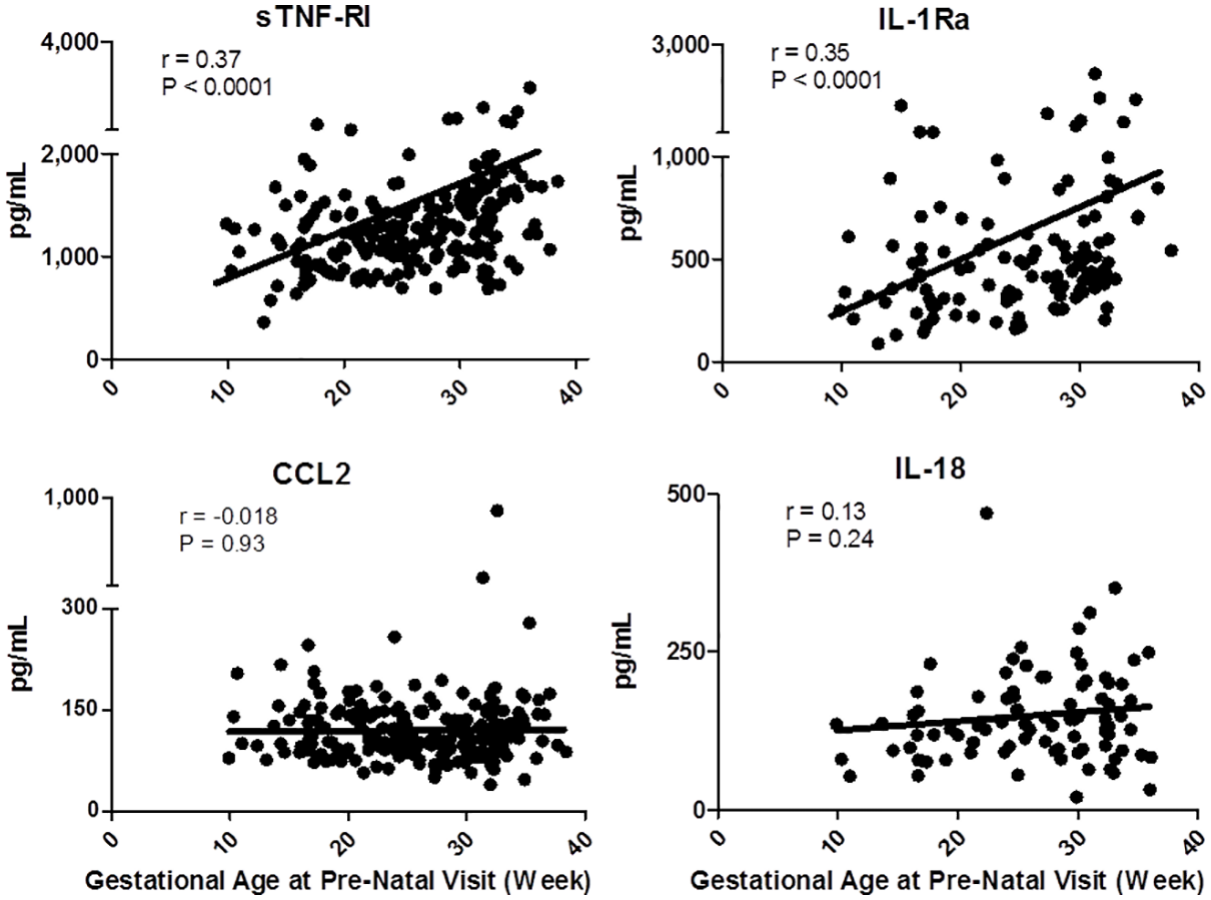
Relationships between gestational age and intensity of anti-inflammatory or pro-inflammatory plasma biomarker expression. Spearman regression analyses are shown.

Finally, to determine if the *intensity* of anti-inflammatory phenotypic changes are altered with the progression of pregnancy, we assessed relationships between plasma biomarker concentrations and gestational age. sTNF-RI, IL-1Ra (Fig 6) and sTNF-RII (not shown) all exhibited increasingly intense *in vivo* expression with increased gestational age. Conversely, the decrease in pro-inflammatory cytokine expression (Fig 6: CCL2 and IL-18; data not shown: CXCL8, CXCL10, IL-6 and TNFα) was constant and exhibited no relationship with increasing gestational age. Thus, the shift towards an increasingly anti-inflammatory bias intensified with increasing gestational age during healthy pregnancy.

## Discussion

Although most pregnancies lead to healthy infants, prior research has focused heavily on diseases of pregnancy. Here, maternal health was examined in 251 healthy pregnant women over up to three years. The data reveal that at least as early as the second trimester, women exhibit increasingly strong constitutive expression of anti-inflammatory mediators and reduced expression of many, but not all, cytokines linked to host defense and inflammatory immune capacity. This transient anti-inflammatory phenotype intensifies with increasing gestational age and is self-resolving by one year postpartum.

Our primary focus here is maternal health and the systemic, constitutive innate immune status exhibited by women during pregnancy. This is not an investigation of factors which enable pregnancy to become established, to proceed to term, or to respond to acute viral or bacterial infection. There is a longstanding lack of agreement regarding constitutive innate immune status during a healthy pregnancy. Small cohorts of healthy pregnant women are incorporated in most studies that examine diseases of pregnancy. In those studies, cross-sectional comparisons are drawn with a broad range of difficult pregnancies, rather than explicitly using well-powered longitudinal study designs to examine the status of healthy women during vs. post-pregnancy. Not surprisingly, such studies do not provide a clear consensus. One school argues that systemic immune status *in vivo*, unlike immune status at the maternal/fetal interface, reflects a generalized mild inflammatory response [70–73]. Others argue for the dominance of anti-inflammatory responses [74, 75]. These apparent contradictions may be due to cross-sectional study designs, cohorts of relatively small size, the resulting impact of subject to subject variability, and reliance on a small number of biomarkers (often one) to assess innate immune bias *in vivo*. For example, two of the largest longitudinal studies available [76, 77] are composed of 48 and 21 healthy women respectively who are followed through pregnancy and postpartum. Many studies involve different ethnicities: Kraus included a 94% Hispanic/Black population [76], while Szarka utilized a Caucasian population [78]. As pointed out more than two decades ago [79], due to high inter-subject variability inherent to any outbred population, a longitudinal, matched study design involving a large n is logistically challenging, but highly important.

This research has important caveats. Firstly, because a longitudinal, multi-year study design was selected to achieve increased sensitivity, it was not socially or logistically feasible to recruit individuals prior to an anticipated pregnancy. For this reason, pregnant women were compared with their immune status at one year and, for a subset, three years postpartum. Pre-pregnancy immune status could not be directly determined. Secondly, a potential confounder that cannot be ruled out is the impact of specific environmental antigens and pathogens on these findings. Women with complicated pregnancies with known linkages to altered innate immunity (i.e. preeclampsia) are excluded from this study. Among the women studied, clinical assessments at each blood draw excluded women with active transient URT or GI infections. The data reported above, characterizing the constitutive *in vivo* phenotype of healthy normal pregnancy positions us to better assess the impact of environmental and genetic influences in pregnancy in subsequent studies. A third caveat is that the impact of menstrual stage postpartum at sampling could influence some values obtained. This underlines the need for sufficient power because the impact of such variables is increasingly reduced as the number of individuals examined increases.

A potential confounder to interpretation of the data obtained was day-to-day stability of biomarker expression within a given individual. We found this to have minimal impact because, as shown in Fig 4, and as was previously reported in short-term studies conducted in men and non-pregnant women [76, 80], systemic levels of most biomarkers are remarkably stable across time (days-weeks) in healthy individuals.

The immunological and clinical implications that result from the increasingly pronounced anti-inflammatory phenotype that developed over the course of healthy pregnancy remains speculative. Extensive literature demonstrates associations between pro-inflammatory or type 1 cytokines and recurrent spontaneous miscarriage or preterm delivery. Exogenous TNFα and IFN inhibit the outgrowth of human trophoblasts *in vitro* and induce apoptosis of human villous trophoblast cells. Murine studies demonstrate that administration of pro-inflammatory cytokines (i.e. TNFα, IFN, IL-2) into pregnant mice causes increased abortion. Conversely, administration of IL-10 prevents fetal wastage in abortion-prone mice [81, 82]. Thus, there is consensus that controls on excessive pro-inflammatory innate responses are essential for successful pregnancy [75].

These data may stand in contrast to *in vivo* expression of acute phase proteins during healthy pregnancy. Multiple studies have found increased plasma/serum CRP levels vs. non-pregnant controls [83–86]. The extent to which changes in this largely IL-6 and TNFα driven biomarker of inflammation are due to activation of classical innate immune responses or other cells (i.e. adipocytes or necrotic processes associated with placenta ageing) is under active investigation [83, 87, 88]. Interestingly, several groups have found undetectable changes or decreases in CRP over the course of healthy pregnancy [83, 89–92]. This inconsistency, and the many pregnancy independent factors (i.e. obesity) that can influence CRP levels, underlines a need for caution and continued research prior to drawing mechanistic conclusions about the role any of these mediators play in successful conclusion of pregnancy.

In this study, we do not address putative differences in maternal immune responses upon infection *in vivo* or acute *in vitro* activation. Studies examining *in vitro* responses to various pattern recognition receptor (PRR) ligands are currently underway as a complementary approach to understanding changes in maternal health that can occur during pregnancy.

With increasing attention given to defining what constitutes a healthy pregnancy [93], this study provides valuable insight into systemic innate immune changes that result in expression of an increasingly intense anti-inflammatory phenotype *in vivo* during pregnancy. It underlines the need for further characterization of what constitutes a successful environment for healthy human pregnancy.

## Supporting information

S1 File Raw duplicates and triplicates from immunological assays performed. (XLSX)
